# Autophagy–Lysosome Pathway Dysfunction in Neurodegeneration and Cancer: Mechanisms and Therapeutic Opportunities

**DOI:** 10.3390/ijms27010366

**Published:** 2025-12-29

**Authors:** Mingyang Du, Yang Yu, Jiachang Wang, Cuicui Ji

**Affiliations:** Department of Biology, College of Chemistry and Life Science, Beijing University of Technology, 100124 Beijing, China

**Keywords:** autophagy, lysosome, neurodegenerative disease, cancer

## Abstract

The autophagy–lysosome system is a master regulator of cellular homeostasis, integrating quality control, metabolism, and cell fate through the selective degradation of cytoplasmic components. Disruption of either autophagic flux or lysosomal function compromises this degradative pathway and leads to diverse pathological conditions. Emerging evidence identifies the autophagy–lysosome network as a central signaling hub that connects metabolic balance to disease progression, particularly in neurodegenerative disorders and cancer. Although cancer and neurodegenerative diseases exhibit seemingly opposite outcomes—uncontrolled proliferation versus progressive neuronal loss—both share common mechanistic foundations within the autophagy–lysosome axis. Here, we synthesize recent advances on the roles of autophagy and lysosomal mechanisms in neurodegenerative diseases and cancer, especially on how defects in lysosomal acidification, membrane integrity, and autophagosome–lysosome fusion contribute to toxic protein accumulation and organelle damage in Alzheimer’s and Parkinson’s diseases, while the same machinery is repurposed by tumor cells to sustain anabolic growth, stress tolerance, and therapy resistance. We also highlight emerging lysosome-centered therapeutic approaches, including small molecules that induce lysosomal membrane permeabilization, nanomedicine-based pH correction, and next-generation protein degradation technologies. Finally, we discuss the major challenges and future opportunities for translating these mechanistic insights into clinical interventions.

## 1. Introduction

Cellular homeostasis critically relies on the regulated degradation and recycling of intracellular materials through autophagy, a conserved catabolic process that directs cytoplasmic cargo to lysosomes [1,2]. Beyond its fundamental housekeeping functions, autophagy participates in diverse physiological processes, such as the maintenance of intracellular homeostasis, adaptation to nutrient deprivation and metabolic stress, regulation of cell development and differentiation, and the modulation of aging [3,4,5]. Dysfunction of autophagy has been linked to numerous pathological conditions, such as neurodegenerative disorders, cancer, infection, and metabolic diseases, underscoring its essential role in health and disease [6,7].

Lysosomes, acidic organelles equipped with over 60 hydrolases, serve as the terminal degradative compartment for both the autophagy–lysosome and endolysosome pathways [8,9]. Through these interconnected routes, cells eliminate protein aggregates, clear dysfunctional mitochondria, and metabolize extracellular material, thereby providing raw materials for the biosynthetic processes and energy production within the cells. Lysosomal dysfunction has emerged as a convergent hallmark of multiple human diseases [10,11]. Defects in lysosomal acidification, hydrolase activity, or membrane integrity disrupt cellular quality control mechanisms, causing the accumulation of toxic substrates and metabolic disturbances [11,12]. This lysosomal impairment drives the pathogenesis of over 70 monogenic lysosomal storage disorders and contributes to complex diseases, including Alzheimer’s disease, Parkinson’s disease, and various cancers [13,14]. The convergence of these disease mechanisms on lysosomal dysfunction has positioned lysosomes as attractive therapeutic targets.

Emerging research has established that autophagy and lysosomes are not merely degradative entities but also pivotal signaling hubs that integrate nutrient sensing, metabolic control, and stress responses. The reciprocal regulation between autophagy and lysosomes underscores their functional interdependence—autophagic activity influences lysosomal number and function, whereas lysosomal efficiency determines the completion of autophagic flux. Perturbation in this cross-regulatory network is increasingly recognized as a key pathogenic driver in a spectrum of diseases. Dysregulation of the autophagy–lysosome system contributes to numerous pathological conditions, particularly neurodegenerative diseases and cancer, which paradoxically represent opposite ends of cellular fate: progressive neuronal death versus uncontrolled proliferation. In neurodegenerative disorders, impaired lysosomal acidification, defective autophagosome–lysosome fusion, and compromised proteolysis result in toxic protein accumulation, organelle dysfunction, and neuronal loss. Conversely, cancer cells exploit the same machinery to sustain anabolic growth, metabolic plasticity, and resistance to therapeutic stress. Despite these contrasting outcomes, both pathological states share convergent molecular mechanisms within the autophagy–lysosome axis, including altered mTOR signaling, defective vesicular trafficking, and aberrant mitophagy.

This review summarizes current advances in the structure and regulation of the autophagy–lysosome system, its integrated role in cellular metabolism, and the consequences of its dysfunction in neurodegenerative diseases and cancer. Recent advances in autophagy and lysosomal biology have catalyzed the development of novel therapeutic strategies. Small-molecule lysosomal modulators, nanomedicines, and technologies for targeted protein degradation show promise in experimental models and clinical trials [15,16]. We highlight recent progress in autophagy–lysosome-targeted therapeutic strategies—including small-molecule modulators, nanomedicine-based pH correction, and emerging protein degradation technologies—and discuss future perspectives for translating mechanistic insights into precision medicine.

## 2. Overview of Lysosome

**Figure 1:**
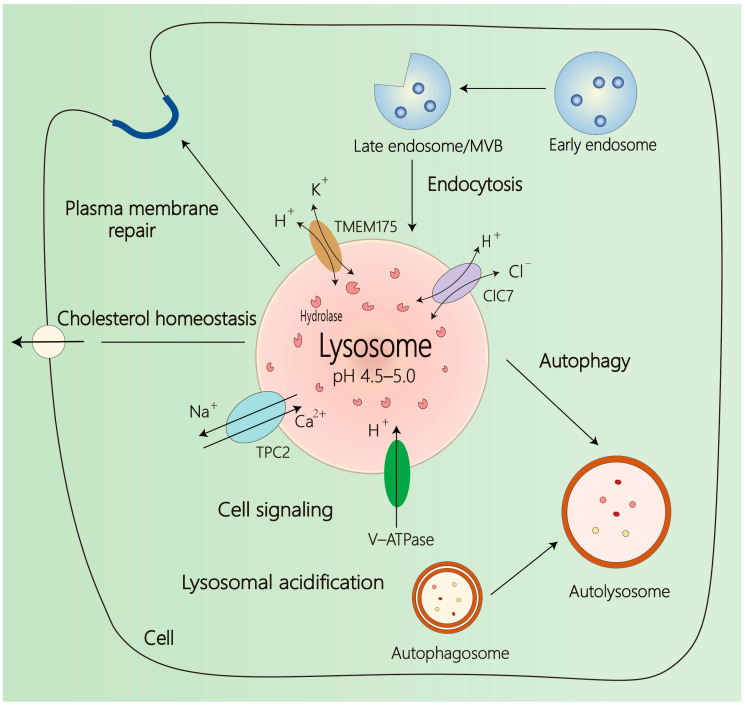
Lysosomal functions and dynamics. Lysosomes serve as central hubs for cellular degradation and metabolic signaling. They maintain an acidic luminal environment (pH ≈ 4.5–5.0) that optimizes the activity of diverse hydrolytic enzymes. Proper acidification and ionic balance are maintained by the coordinated activity of membrane proteins, including V-ATPase, TMEM175, CLC7, and Na^+^/Ca^2+^ exchangers. Extracellular cargos are delivered to lysosomes through endocytosis, during which early endosomes progressively mature into late endosomes that fuse with the lysosomes. In autophagy, cytoplasmic substrates are targeted to lysosomes via autophagosome–lysosome fusion. Beyond degradation, lysosomes also contribute to cholesterol metabolism, plasma-membrane repair, nutrient sensing, and signal transduction.

Lysosomes are spherical organelles with a single-layered membrane structure, ranging from 0.2 to 0.8 μm in diameter and ubiquitously distributed across nearly all eukaryotic cell types [17]. They contain more than 60 distinct acidic hydrolases—including cathepsins, nucleases, lipases, glycosidases, and phosphatases—which are produced within the endoplasmic reticulum and mature in the Golgi apparatus before lysosomal targeting via mannose-6-phosphate receptor [18]. Individual cells typically contain hundreds of lysosomes that form a dynamic intracellular network essential for cellular homeostasis. Lysosomal degradation is achieved through two major pathways: the endocytic pathway, which delivers extracellular and plasma membrane cargo, and autophagy, which targets intracellular components [17]. Via these convergent routes, lysosomes catabolize damaged organelles and internalized macromolecules into metabolic precursors that are recycled to maintain cellular biosynthetic processes. Disruption of lysosomal function impairs these degradative processes, leading to substrate accumulation, metabolic imbalance, and the onset of numerous diseases (Figure 1).

The degradative capacity of lysosomes depends on their acidic environment (pH 4.5–5.0), which optimally activates resident hydrolases. Maintenance of lysosomal acidity depends on a balance of proton influx and efflux: (1) vacuolar H^+^-ATPase (V-ATPase)-mediated H^+^ pumping and (2) H^+^ leak pathways. V-ATPase is an ATP-driven proton pump whose activity is tightly regulated by nutrient status, subunit composition, and assembly dynamics [11]. Lysosomal H^+^ leak could be mediated by TMEM175 and SLC7A11 [19,20].

Beyond hydrolases and proton pumps, lysosomes contain numerous structural and regulatory proteins critical for their function. These include structural proteins such as LAMP1 (lysosome-associated membrane protein 1), transport mediators such as LAMP2A (lysosome-associated membrane protein type 2A), and ion channels such as CLC7 (chloride channel protein 7) [21,22,23]. Interestingly, CLC7, functioning as an H^+^/Cl^−^ antiporter, maintains lysosomal membrane potential and pH homeostasis by extruding chloride ions (Cl^−^) to counterbalance the positive charge generated by V-ATPase-mediated proton influx [24]. Collectively, these components maintain lysosomal stability, trafficking, and signal transduction, underscoring the lysosome’s role as a central hub for cellular metabolism and homeostasis.

## 3. Overview of Autophagy

**Figure 2:**
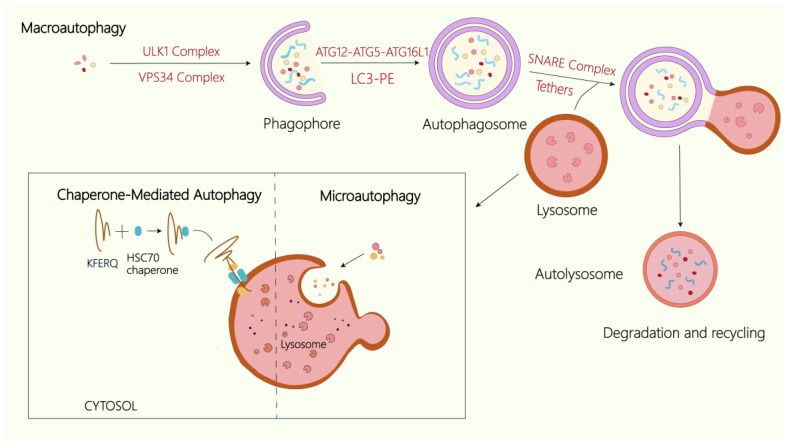
Three types of autophagy. In mammalian cells, the lysosome serves as the terminal degradative compartment for all forms of autophagy. Macroautophagy is initiated by the ULK1/2 complex, whose activity is modulated by upstream nutrient and energy signals through mTORC1 and AMPK. The PI3K complex facilitates phagophore nucleation, while two ubiquitin-like conjugation systems—the ATG12–ATG5–ATG16L1 complex and the LC3–PE system—drive membrane expansion and autophagosome formation. Subsequent fusion of the autophagosome with lysosomes is facilitated by multiple tethering and SNARE complexes, enabling degradation and recycling of cytoplasmic components. In chaperone-mediated autophagy, cytosolic substrates bearing the KFERQ pentapeptide motif are selectively recognized by HSPA8/HSC70 and co-chaperones, delivered to the lysosomal membrane, and translocated into the lumen through LAMP2A for degradation. During microautophagy, cytoplasmic cargos are directly engulfed by the lysosome via membrane invagination or deformation, allowing bulk turnover of cytosolic material.

Autophagy is a conserved degradative process in eukaryotic cells that eliminates aged or damaged organelles and macromolecules within the cytoplasm. While basal autophagy is essential for cellular homeostasis, its dysregulation—either deficiency or hyperactivation—can be pathogenic [25,26,27]. Autophagic dysfunction underlies numerous diseases, frequently resulting from impaired lysosomal degradation and consequent accumulation of undegraded substrates. Based on cargo delivery mechanisms to lysosomes, autophagy can be divided into three major types: microautophagy, macroautophagy, and chaperone-mediated autophagy (CMA) (Figure 2) [25]. Macroautophagy, the most extensively characterized form, involves the generation of double-membrane autophagosomes that sequester cytoplasmic cargo. These autophagosomes subsequently fuse with lysosomes to produce autolysosomes, where cargo is degraded into metabolites such as amino acids and fatty acids, which are then recycled to maintain cellular metabolism and homeostasis [28]. Various kinds of cargo lead to categorizing autophagy into either nonselective or selective forms. The initiation of the selective autophagy pathway is induced by specific cargos, which are engulfed by the autophagosome and directed to lysosomes for targeted degradation [29,30]. Depending on the specific cargos, selective autophagy is divided into various subtypes, such as aggrephagy (protein aggregates), ER-phagy (ER), mitophagy (mitochondria), the Cvt pathway in yeast (prApe1), pexophagy (peroxisomes), and lyophagy (lysosomes) [30]. Importantly, the selectivity of this process is mediated by distinct autophagy receptors that specifically recognize cargos and direct them to the autophagy machinery for degradation. CMA is a highly selective pathway that specifically targets proteins containing a pentapeptide KFERQ motif. During CMA, substrate proteins bearing this motif are recognized by the cytosolic chaperone HSC70 (HSPA8) and its co-chaperones, then delivered to lysosomes, where they bind LAMP2A. This interaction triggers LAMP2A oligomerization, facilitating substrate translocation across the lysosomal membrane [31]. By contrast, microautophagy involves the direct engulfment of cytoplasmic constituents through invagination or protrusive remodeling of the lysosomal membrane [32].

Autophagy requires sophisticated regulatory machinery. In mammalian cells, initiation of macroautophagy involves the ULK1 complex—comprising ULK1, ATG13, ATG101, and FIP200—that assembles in response to cellular stress signals [33]. Under nutrient deprivation, mTOR inhibition coupled with AMPK activation promotes ULK1 phosphorylation, triggering autophagosome biogenesis. The class III phosphatidylinositol 3-kinase (PI3K) complex, containing Beclin 1, VPS34, NRBF2, p150, and ATG14L, subsequently catalyzes PI3P production at nucleation sites [34]. Autophagosome formation involves two ubiquitin-like conjugation systems: the ATG12-ATG5-ATG16L1 and LC3-PE systems. Through sequential reactions catalyzed by ATG7 and ATG10, ATG12 conjugates to ATG5, which then recruits ATG16L1 to form the ATG12-ATG5-ATG16L1 complex essential for isolation/phagophore expansion. Similarly, ATG4 cleaves pro-LC3 to generate LC3-I, which is subsequently lipidated with phosphatidylethanolamine (PE) to produce membrane-bound LC3-II [8,35]. Recruitment of LC3-II by the ATG12-ATG5-ATG16L1 complex drives phagophore elongation and closure, ultimately producing mature double-membrane autophagosomes [36]. Autophagosome–lysosome fusion requires multiple tethering and fusion complexes, including the tethers and SNARE complex [37,38,39]. Following fusion, lysosomal hydrolases degrade the inner autophagosomal membrane and its cargos, releasing amino acids and other metabolites for cellular recycling.

## 4. The Autophagy–Lysosome Axis: An Integrated Degradative Network

All forms of autophagy ultimately converge on the lysosome, where degradation occurs. Lysosomal biogenesis, localization, pH regulation, and enzymatic activity determine the efficiency of autophagic clearance. Conversely, autophagy machinery influences lysosomal formation, positioning, stability, and activity. Together, autophagy and lysosomes form a reciprocal and integrated degradative network essential for cellular quality control and metabolic recycling.

Lysosomes serve as the principal catabolic compartments in mammalian cells. Their dysfunction disrupts autophagic flux, leading to the accumulation of autophagosomes and autolysosomes. In macroautophagy, autophagosome–lysosome fusion is a rate-limiting step in cargo degradation. This process involves multiple molecular components, including SNARE complexes (STX17–SNAP29–VAMP7/8 and STX7–SNAP29–YKT6), which function in a partially redundant manner, as well as tethering factors such as HOPS, EPG5, and PLEKHM1 that bridge autophagosomal and lysosomal membranes [40,41]. Small GTPases, including RAB7, Arl8b, and RAB32, further regulate tethering and fusion [42]. Notably, PLEKHM1 simultaneously binds LC3 on autophagosomes and RAB7/Arl8b on lysosomes, while GTP-bound RAB7 enhances EPG5 recruitment to late endosomes and lysosomes, thereby promoting efficient fusion [43,44].

Autophagy initiation is tightly regulated by the mechanistic target of rapamycin complex 1 (mTORC1), which senses nutrient availability in both the cytoplasm and lysosomes. Under nutrient-rich conditions, amino acids activate Rag GTPases and the Ragulator complex to recruit mTORC1 to lysosomal membranes, where Rheb further activates it [45]. Activated mTORC1 further phosphorylates ULK1 and ATG13 within the initiation complex, thereby suppressing autophagy [46]. Conversely, nutrient deprivation prevents mTORC1 activation, relieving this inhibition and triggering autophagy.

During active autophagy, lysosomal activity and perinuclear clustering increase, facilitating autophagosome–lysosome fusion. Starvation also activates TFEB, a key modulator of both lysosomal biogenesis and autophagy. Under conditions of nutrient sufficiency, TFEB/TFE3 associates with Rag GTPases and remains on the lysosomal surface, where mTORC1-dependent phosphorylation prevents nuclear entry [47]. Upon nutrient deprivation, inactive Rag GTPases fail to recruit mTORC1, leading to TFEB/TFE3 dephosphorylation and nuclear translocation [48]. Within the nucleus, TFEB/TFE3 binds CLEAR (Coordinated Lysosomal Expression and Regulation) motifs to drive expression of lysosomal and autophagy genes, thereby enhancing degradative capacity [49]. TFEB/TFE3 activity is also modulated by additional signaling cascades: STING activation inhibits mTORC1-mediated TFEB phosphorylation, while starvation-induced lysosomal Ca^2+^ release activates the phosphatase calcineurin, which further dephosphorylates TFEB and promotes its nuclear import [50,51].

As the principal degradative organelles, lysosomes are susceptible to damage from various stressors. Damaged lysosomes are selectively removed by lysophagy. When repair mechanisms fail, galectin proteins such as Gal3 recognize exposed luminal glycans on ruptured lysosomes and recruit the E3 ligase TRIM16 [52]. TRIM16 acts as both an autophagy receptor—assembling ULK1 and Beclin1 complexes—and a regulator that mediates K63-linked ubiquitination of ULK1 and Beclin1 to facilitate autophagic degradation. In a galectin-independent mechanism, exposure of LAMP2 on damaged lysosomes recruits WDFY1, which binds LAMP2 and assembles the CUL4A–DDB1–WDFY1 complex. This complex mediates K48-linked ubiquitination of LAMP2, enabling recruitment of downstream autophagy machinery to complete lysosomal clearance [53].

## 5. Autophagy–Lysosome Pathway Dysfunction in Diseases

**Figure 3:**
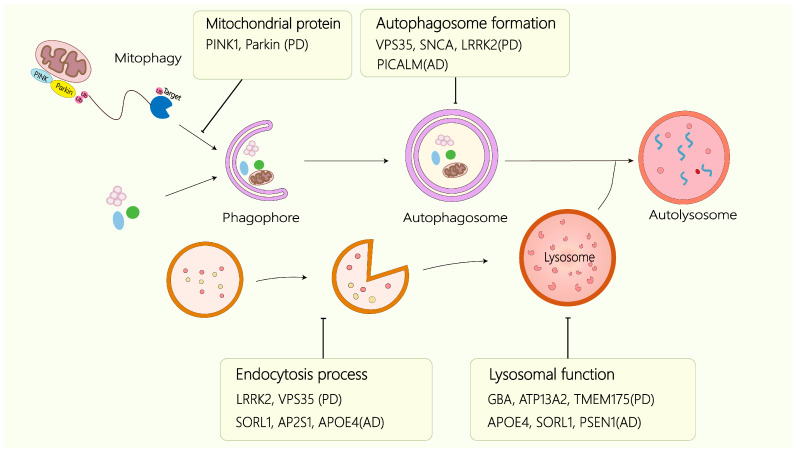
Molecular defects in the autophagy–lysosomal pathway associated with Alzheimer’s and Parkinson’s diseases. A subset of genes—including AP2S1, SORL1, APOE4, PSEN1, TMEM175, LRRK2, VPS35, ATP13A2, GBA, SNCA, PICALM, PINK1, and Parkin—has been implicated in Alzheimer’s or Parkinson’s disease. Those genes are involved in the autophagy–lysosomal pathway and participate at distinct stages of the autophagic process, where their functional disruption contributes to disease-specific neurodegenerative mechanisms. The proposed sites of action for each gene corresponding to the particular pathological context of the associated disorder are indicated.

Lysosomes are central to cellular metabolism and homeostasis. Dysregulation of lysosomal activity results in the accumulation of undegraded metabolites—including proteins and lipids—as well as disruption of autophagy, endocytosis, and other essential pathways. Such defects are closely associated with the onset and progression of diverse diseases, most notably neurodegenerative disorders and cancer. Neurodegenerative diseases frequently present with intracellular protein inclusions or extracellular aggregates of misfolded proteins. Impairments in protein trafficking or degradation drive this accumulation, and lysosomal degradation represents a crucial pathway for maintaining proteostasis. Mounting evidence indicates that the pathogenesis of neurodegenerative diseases, such as Alzheimer’s and Parkinson’s diseases, involves lysosomal disruption and dysfunction of autophagy–lysosomal and endolysosomal pathways (Figure 3). Beyond neurodegeneration, lysosomes also play pivotal roles in tumor initiation, progression, drug resistance, and therapeutic response, making them important targets for cancer research and treatment.

### 5.1. Neurodegenerative Diseases

#### 5.1.1. Alzheimer’s Disease

Alzheimer’s disease (AD) is an age-related neurodegenerative disorder characterized by the extracellular accumulation of amyloid-β (Aβ) plaques and the intracellular formation of neurofibrillary tangles (NFTs) [54]. As Aβ accumulates, it aggregates into amyloid plaques within the brain, leading to neuronal apoptosis. NFTs are composed of hyperphosphorylated microtubule-associated tau protein. Phosphorylation at key residues such as Thr181 and Thr217 markedly increases tau’s resistance to lysosomal proteolysis through its proline-rich domain, and this resistance intensifies with elevated lysosomal pH [55]. Impaired lysosomal function is a major cause of protein homeostasis imbalance. Although the precise molecular mechanisms of AD remain incompletely understood, the accumulation of Aβ and NFTs is closely linked to degradation defects arising from lysosomal dysfunction. Ching-Chieh Chou et al. reported that lysosomal dysfunction in AD-induced transdifferentiated neurons derived from human fibroblasts was more severe than that observed in aged control neurons. They found that lysosomal damage not only disrupts protein homeostasis but also exacerbates neuroinflammatory responses. In particular, AD neurons with lysosomal impairment exhibited elevated secretion of inflammatory cytokines, including IL-6, IL-15, and CCL2 [56].

Several AD-related genes—including PSEN1, SORL1, and APOE4—directly affect degradation pathways by altering lysosomal function. PSEN1 encodes presenilin-1 (PS1), and its mutation or knockout results in defects in the vacuolar V-ATPase complex, disrupting lysosomal acidification and impairing Aβ degradation [57]. Mutations in PSEN1 and APP also lead to defects in the endolysosomal and autophagic systems [58]. Mutations in SORL1, a major cause of autosomal dominant AD, result in abnormal lysosomal morphology and dysfunction, leading to enlarged endosomes and impaired autophagy [59]. Reactive intermediates of APOE4 can bind tightly to phospholipid bilayers and insert into lysosomal membranes, causing hydrolase leakage and apoptosis. The APOE4 variant also enhances APP endocytosis and disrupts endolysosomal recycling [60].

Genes such as RAB5 and AP2S1 also regulate substrate degradation by modulating lysosome-associated pathways. Hyperactivation of RAB5 in AD mouse models causes proteolytic and autophagic defects, enlarges early endosomes, and enhances endocytosis. AP2S1 loss reduces Aβ levels, while its overexpression increases APP and Aβ production. AP2S1 deficiency promotes APP transport from late endosomes to lysosomes and enhances autophagosome–lysosome fusion [61]. In addition, ROCK1, a serine/threonine kinase, regulates lysosomal biogenesis and acidification. Decrease in ROCK1 reduces the levels of Aβ and tau in AD mice. Overexpression of ROCK1 reduces lysosome number and disrupts the acidic microenvironment required for optimal enzymatic activity [62]. Collectively, lysosomes have emerged as one of the most promising therapeutic targets for AD drug development.

#### 5.1.2. Parkinson’s Disease

Parkinson’s disease (PD), second in prevalence to Alzheimer’s disease, is marked by the progressive degeneration of dopaminergic neurons within the substantia nigra pars compacta and the presence of Lewy bodies. Lewy bodies consist primarily of aggregated α-synuclein along with fragmented membranes and organelles [63]. Several lysosome-associated genes have been identified as PD risk factors, including GBA, TMEM175, CTSB, and ATP13A2. GBA encodes β-glucocerebrosidase (GCase), a lysosomal enzyme essential for normal lysosomal activity and α-synuclein degradation [64]. TMEM175 forms a proton-selective channel in the lysosomal membrane that regulates luminal pH, membrane potential, and organellar fusion. Loss of TMEM175 function results in unstable lysosomal pH, impaired autophagosome–lysosome fusion, and enhanced α-synuclein aggregation [19]. Recent studies have identified novel TMEM175 activators with promising therapeutic potential, including DCY1020, DCY1040, and TUG-891 [65]. The utility of TMEM175 modulators for personalized PD treatment has been validated in Chinese PD cohorts, where multiple clinical mutations correlate with reduced TMEM175 channel activity. Importantly, compounds DCY1040 and TUG-891 effectively restored lysosomal acidification in patient-derived samples harboring TMEM175 variants, demonstrating the potential of small-molecule TMEM175 activators for precision medicine approaches [65]. CTSB, a lysosomal cysteine protease, is required for α-synuclein degradation, and its deficiency impairs proteolytic clearance [66]. Mutations in ATP13A2, encoding a lysosomal ATPase, cause juvenile-onset familial PD through disrupted lysosomal acidification and compromised degradative capacity [67].

Autosomal dominant PD genes—including SNCA, PINK1, Parkin, LRRK2, and VPS35—also affect the autophagy–lysosomal axis. SNCA, the first identified PD gene, encodes α-synuclein; its mutations increase α-synuclein production and aggregation, disrupting proteostasis. Wild-type α-synuclein is normally degraded by both macroautophagy and CMA [68]. When lysosomal function is compromised, α-synuclein clearance fails. Notably, α-synuclein functions both as an autophagy substrate and regulator—its overexpression inhibits autophagosome formation by suppressing ATG9 localization [69]. PINK1-Parkin-mediated mitophagy is the most described ubiquitin-dependent mitophagy pathway. When mitochondria are healthy in cells, PINK1 is imported into mitochondria and degraded rapidly, while Parkin remains in an inactive state within the cytosol. Upon mitochondrial damage, the import of PINK1 into the mitochondrial matrix is impeded. Consequently, PINK1 accumulates on the outer mitochondrial membrane (OMM). PINK1 subsequently activates Parkin and promotes its translocation to the surface of damaged mitochondria. Once Parkin translocates to mitochondria, it ubiquitinates various OMM proteins, thereby recruiting autophagy adaptors including p62, NDP52, and OPTN, leading to the clearance of damaged mitochondria by mitophagy [70]. LRRK2 modulates autophagy bidirectionally: wild-type LRRK2 enhances autophagic flux [68], whereas mutant LRRK2 binds LAMP2A, impeding CMA and blocking degradation of both LRRK2 itself and its substrates. LRRK2 also participates in endocytic trafficking by mediating protein transport from endosomes to the trans-Golgi network [71]. VPS35 facilitates retrograde transport from endosomes to the Golgi apparatus and regulates the trafficking of cathepsin D, LAMP2A, and ATG9A, thereby coordinating autophagy and lysosomal processing [72]. The PD-associated VPS35[D620N] mutation activates LRRK2 kinase and promotes RILPL1 binding to the lysosomal membrane protein TMEM55B, ultimately disrupting lysosomal homeostasis [73].

#### 5.1.3. Huntington’s Disease

Huntington’s disease (HD) is caused by the expansion of CAG trinucleotide repeats in the HTT gene, which encodes huntingtin protein, resulting in the production of mutant huntingtin (mHTT). HD pathogenesis involves profound dysfunction of the autophagy–lysosomal system. mHTT undergoes misfolding to form insoluble aggregates whose toxicity drives disease progression. Compared to wild-type protein, mHTT exhibits enhanced secretion [30], accumulates throughout the central nervous system, and exerts pathogenic effects via multiple mechanisms [74].

The autophagy–lysosomal pathway is impaired at multiple levels in HD. mHTT disrupts normal lysosomal distribution, causing perinuclear clustering [75]—a primary site of mTORC1 activation—thereby increasing mTORC1 activity. Additionally, autophagosomes undergo premature fusion with lysosomes. Transcriptomic analysis of striatal neurons reprogrammed from HD patient fibroblasts revealed downregulation of autophagy–lysosome pathway genes compared to healthy controls, consistent with functional measurements showing reduced autophagic flux [76]. Supporting this finding, recent work demonstrated that the transcription factor TFEB contains a prion-like domain (PrLD) that mediates its co-aggregation with mHTT. This sequestration disrupts TFEB activation in cellular reporter systems. The presence of empty autophagosomes in HD patient cells indicates defects in cargo recognition or engulfment [77]. Accumulated mHTT also sequesters Beclin1, further suppressing autophagy initiation.

#### 5.1.4. BPAN

β-propeller protein-associated neurodegeneration (BPAN) is a subtype of neurodegeneration with brain iron accumulation (NBIA) caused by mutations in WDR45 [78,79]. BPAN patients typically present with developmental delay in early childhood and develop spasticity and generalized dystonia in adulthood [80]. WDR45 belongs to the WIPI protein family, which comprises WIPI1, WIPI2, WDR45B/WIPI3, and WDR45/WIPI4. WDR45 and WDR45B regulate autophagosome–lysosome fusion through interaction with the tethering protein EPG5 [81,82]. Recent studies demonstrate that WDR45 mutations impair ferritinophagy, thereby disrupting iron recycling [83]. WDR45 mutations also compromise lysosomal function, as evidenced by reduced levels of lysosomal markers LAMP1 and LAMP2, along with defective lysosomal activity, in fibroblasts from BPAN patients [84].

### 5.2. Cancer

Cancer is a genetic disease driven by both environmental carcinogens and intrinsic factors. It is characterized by uncontrolled cell proliferation supported by reprogrammed metabolic activity [85]. Autophagy functions as a double-edged sword in cancer: during tumor initiation, it preserves genomic stability and suppresses transformation, whereas in established tumors, it sustains growth by providing metabolic substrates under nutrient stress [86].

Autophagy is a highly adaptive process that allows cells to cope with diverse forms of stress, including protein aggregation, organelle damage, and redox imbalance. By eliminating dysfunctional mitochondria and oxidative byproducts, autophagy mitigates DNA damage and reduces mutational burden. Accumulating evidence indicates that autophagy functions as a tumor-suppressive process during the initial phases of tumorigenesis. The connection between autophagy and cancer suppression was first revealed through investigations of Beclin 1 in breast cancer, establishing a mechanistic link between autophagic regulation and oncogenesis [87]. Subsequent work demonstrated that heterozygous loss of Beclin1 in mice leads to spontaneous tumorigenesis in the lung, liver, and lymphoid tissues, providing direct evidence that autophagy constitutes a fundamental mechanism of cell growth control and tumor suppression [88]. Other core autophagy genes, including ATG5 and ATG7, are also implicated in cancer. Reduced expression of ATG5 and ATG7 has been reported in melanoma, while loss of ATG7 promotes hepatic tumor formation [89]. As malignancies advance, additional autophagy-related genes or modulators of ATG proteins frequently acquire mutations or become inactivated, allowing malignant cells to evade autophagy-mediated suppression. In gastric and colorectal carcinomas, several ATG genes containing mononucleotide repeats (ATG2B, ATG5, ATG9B) exhibit frameshift mutations, suggesting that disruption of the autophagic process may drive tumor progression [90].

Mitophagy plays a dual role in tumor development, acting either as a tumor-suppressive process or as a driver of tumor progression depending on the cellular and metabolic milieu. It preserves mitochondrial quality by selectively removing damaged organelles, thereby maintaining metabolic homeostasis and supporting tumor cell survival under stress conditions such as hypoxia and nutrient deprivation within the tumor microenvironment. This adaptive mitophagy protects cancer cells from metabolic stress and contributes to tumor progression. On the other hand, by eliminating dysfunctional mitochondria and reducing the accumulation of reactive oxygen species (ROS), mitophagy can prevent oxidative DNA damage and suppress tumor initiation [91,92]. Mechanistically, PINK1 and PARK2 (Parkin) play central roles in this process. These proteins restrain pancreatic tumorigenesis by modulating mitochondrial iron-dependent immunometabolic pathways. Loss of PINK1 or PARK2 expression accelerates the progression of KRAS-driven pancreatic cancer [93]. Deletions or reduced expression of PINK1 have also been observed in multiple human brain tumors, including glioblastoma, and correlate with poor clinical prognosis. Functionally, PINK1 acts through FOXO3a-mediated suppression of ROS to restrain tumor proliferation. In glioblastoma, loss of PINK1 enhances the Warburg effect by promoting ROS-dependent stabilization of hypoxia-inducible factor-1α (HIF-1α) and reducing the activity of pyruvate kinase M2 (PKM2)—two key regulators of aerobic glycolysis—thereby facilitating metabolic reprogramming that supports malignant growth [94]. Collectively, these findings highlight mitophagy as a critical metabolic checkpoint whose dysregulation can tip the balance between tumor suppression and tumor promotion. Under hypoxic conditions, HIF-1α induces BNIP3-mediated autophagy to maintain cellular energy homeostasis. BNIP3 triggers mitophagy to prevent the accumulation of dysfunctional mitochondria and limit excessive ROS production, thereby promoting breast tumor survival and adaptation [95].

Paradoxically, as the tumor microenvironment develops, autophagy can be co-opted by cancer cells to withstand nutrient deprivation, hypoxia, and therapeutic stress, thereby facilitating tumor progression. For example, activating mutations in RAS, which frequently occur in diverse cancers, enhance tumor proliferation and survival while increasing the cellular demand for energy and biosynthetic precursors. Through self-digestion, autophagy compensates for limited nutrient availability, maintaining metabolic homeostasis and supporting continued tumor growth. This dependency renders certain RAS-driven cancers “autophagy-addicted,” as their progression is markedly impaired in the absence of functional autophagy. Indeed, studies in adult mice have demonstrated that systemic ablation of Atg7 leads to marked regression of KRAS-driven tumors, highlighting the essential role of autophagy in maintaining oncogenic RAS activity [96]. In cancer cells, lysosomes become enlarged and more numerous, accompanied by altered expression of lysosomal enzymes. By modulating growth factor signaling and nutrient availability, lysosomes tightly regulate tumor cell proliferation. To sustain rapid proliferation, tumor cells depend on lysosomes for metabolic adaptation—degrading intracellular components through autophagy and importing extracellular proteins via macropinocytosis to generate amino acids and other biosynthetic precursors [14]. To meet high metabolic demands, cancer cells dynamically adjust lysosomal number, localization, and activity. These adaptations are associated with upregulation of lysosomal and lysosome-associated proteins, such as catalases, glycosidases, and kinesins. Several cancer types—including pancreatic adenocarcinoma, renal cell carcinoma, melanoma, and breast cancer—exhibit overexpression of MIT/TFE transcription factors, which enhance lysosomal gene expression. Such alterations promote cancer cell proliferation, invasion, and resistance to radio- and chemotherapy [97].

As central regulators of nutrient sensing and metabolic signaling, lysosomes influence tumor growth primarily through activation of mTORC1. In many cancers, mTORC1 is hyperactivated on the lysosomal surface, promoting cell proliferation. Loss of tumor suppressors such as PTEN and p53 further amplifies mTORC1 signaling. Elevated protein turnover in tumor cells causes excessive iron accumulation within lysosomes, leading to lysosomal membrane permeabilization (LMP), loss of membrane integrity, and aberrant cell division [98,99]. Additionally, lysosomal cathepsins are often overexpressed and secreted into the extracellular space, where they degrade the surrounding matrix and facilitate tumor invasion [100]. Recent studies have identified the lysosomal autophagy receptor CCDC50 as a key regulator of lysosomal quality control in cancer. CCDC50 recognizes and promotes the autophagic clearance of damaged lysosomes, maintaining redox balance and supporting tumor cell survival. It is highly enriched in melanoma cells and tissues, with expression further elevated in metastatic samples. High CCDC50 expression correlates with increased malignancy, metastasis, and poor overall survival in melanoma patients. Silencing CCDC50 significantly suppresses melanoma cell proliferation, invasion, and metastasis, while improving survival in mouse models, underscoring its tumor-promoting function and potential as a therapeutic target [101].

Overall, lysosomal dysfunction and altered autophagy are closely linked to tumorigenesis. Given their central role in metabolic regulation and cell survival, lysosomes represent promising therapeutic targets. Current lysosome-targeted cancer therapies primarily act by disrupting lysosomal acidity or membrane stability, thereby suppressing tumor growth and enhancing treatment efficacy.

### 5.3. The Interaction Between Neurodegenerative Diseases and Cancer

Neurodegenerative diseases and cancer are both age-related disorders that represent opposing ends of the cellular fate spectrum. Cancer is defined by uncontrolled cellular proliferation, invasion, and metastasis, whereas neurodegenerative disorders such as AD are marked by progressive neuronal dysfunction, cell loss, and structural atrophy. Tumorigenesis arises from the cumulative effects of genetic mutations, epigenetic dysregulation, and complex interactions between intrinsic and extrinsic determinants, while neurodegeneration results from the gradual failure of neuronal homeostasis, culminating in cognitive, motor, and behavioral decline. Substantial epidemiological evidence supports an inverse comorbidity between them: cancer survivors exhibit a significantly reduced risk of developing AD or PD, and conversely, patients with neurodegenerative diseases have a lower incidence of malignancy [102,103,104]. This bidirectional protection suggests that cancer and neurodegeneration are inversely regulated to determine cell survival or death.

Molecular studies reveal that several signaling cascades are shared in these disorders. For example, the PI3K/Akt/mTOR axis, which promotes proliferation and metabolic adaptation in tumors, is frequently downregulated in AD, where its inhibition impairs synaptic plasticity and enhances tau hyperphosphorylation [105,106]. Similarly, the tumor suppressor p53 exhibits a dual nature: loss-of-function mutations in cancer disrupt cell-cycle arrest and apoptosis, promoting tumorigenesis, whereas excessive activation of p53 in neurons induces apoptosis and synaptic dysfunction [107,108,109]. Intracellular Aβ42 accumulation in AD directly activates the p53 promoter, triggering p53-dependent neuronal death [110]. APOE4 also exerts tumor-suppressive effects—its expression correlates with enhanced antitumor immunity, reduced melanoma progression, and inhibition of canonical Wnt signaling, underscoring its context-dependent biological function [111,112].

Several genes implicated in neurodegenerative diseases also participate in oncogenic or tumor-suppressive networks. α-synuclein, PTEN, PINK1, DJ-1 (PARK7), LRRK2, and tau (MAPT) are critical for neuronal integrity, yet their mutations or aberrant expression are also observed in various cancers [113]. Among them, DJ-1 exemplifies this duality: it is a recessively inherited causative gene for early-onset PD, where its deficiency destabilizes Nrf2, weakens antioxidant defenses, and increases neuronal susceptibility to oxidative stress and apoptosis [114,115]. Conversely, DJ-1 is overexpressed in multiple malignancies—including lung, breast, colon, liver, and melanoma—where it activates Nrf2- and Akt-dependent prosurvival signaling, facilitating tumor growth and metastasis [102]. A particularly striking point of convergence lies in mitophagy, which is a central determinant of cellular fate, balancing survival and death. Key neurodegenerative disease-related genes such as PINK1, Parkin, and DJ-1 directly regulate this process. Dysregulation of mitophagy leading to the accumulation of defective mitochondria, a hallmark shared by both cancer and NDDs. In tumors, mitophagy is often suppressed or co-opted to maintain metabolic flexibility under stress, while in neurodegenerative diseases, its impairment leads to neuronal energy failure and degeneration. Mitochondrial autophagy receptors and adaptors—including PINK1, Parkin, BNIP3, BNIP3L/NIX, and p62/SQSTM1—as well as the upstream signaling pathways governing mitophagy, are commonly altered in both contexts [91]. Thus, while oxidative stress, inflammation, and cell death are shared pathological features, mitophagy emerges as the biological intersection where the mechanisms of cancer and neurodegeneration converge.

## 6. Autophagy–Lysosome-Targeted Therapeutic Strategies

The autophagy–lysosome system constitutes a conserved degradative pathway essential for maintaining intracellular homeostasis. Through the coordinated degradation and recycling of macromolecules and organelles, this dynamic process enables cells to adapt to metabolic stress and maintain neuronal and tissue integrity. Increasing evidence indicates that pharmacological agents targeting components of the autophagic machinery or lysosomal function hold considerable promise as therapeutic interventions for neurodegenerative diseases and cancer (Table 1).

Enhancement of the autophagy–lysosome pathway has shown therapeutic promise across multiple diseases. Overactivation or dysregulation of the autophagic process can itself drive cell death. Several mTOR pathway inhibitors—such as rapamycin, temsirolimus, everolimus, ridaforolimus, and GDC-0980—exert antineoplastic effects through enhanced autophagy-mediated cytotoxicity. Among these, temsirolimus has gained FDA approval for the management of advanced renal cell carcinoma, whereas everolimus, ridaforolimus, and GDC-0980 are currently being evaluated in clinical trials for various tumor types [116,117]. Moreover, metabolic modulators and naturally derived compounds have attracted attention for their roles in autophagy regulation. Metformin, a widely prescribed antidiabetic drug, activates autophagy via the AMPK pathway, contributing to its tumor-suppressive properties [118,119]. Similarly, curcumin, a bioactive phytochemical, has been shown to induce autophagy and suppress glioblastoma proliferation [120]. Notably, in the APP/PS1 transgenic mouse model of AD, metformin-induced activation of chaperone-mediated autophagy (CMA) substantially attenuates amyloid-β plaque deposition in the brain. This modulation of CMA restores both molecular and behavioral deficits characteristic of Alzheimer’s pathology, underscoring its therapeutic potential in mitigating neurodegeneration [121].

In addition, celastrol also induces autophagy in certain cancers, including gastric and renal cell carcinomas, thereby suppressing tumorigenesis and progression [122]. TFEB functions as a central transcriptional regulator and controls autophagosome formation and lysosome-related gene expression. In AD, reduced TFEB activity contributes to impaired autophagic clearance. Celastrol, a small-molecule TFEB activator, enhances autophagy and lysosomal biogenesis, reducing phosphorylated tau aggregates and offering potential as an AD therapeutic [123]. Similarly, trehalose, a food-grade disaccharide, enhances lysosomal degradation and autophagy by inhibiting TFEB phosphorylation, thereby improving pathologies in HD and PD [124]. DNA damage and excessive PARP1 activation are hallmarks of neurodegeneration. While PARP1 initially facilitates DNA repair, its overactivation impairs autophagy by promoting cytoplasmic translocation of TFEB and disrupting lysosomal function [125]. Thus, PARP1 inhibitors show promise for neurodegenerative disorders. The thioredoxin (Trx) system represents another therapeutic target. The natural compound isowalsuranolide (Hdy-7), isolated from Walsura yunnanensis, inhibits Trx reductase (TrxR1/2), causes reactive oxygen species accumulation, p53 pathway activation, and induction of TFEB/TFE3-dependent lysosomal biogenesis and autophagy via the SESN2–mTOR axis [126]. Notably, paclitaxel-resistant tumor cells remain sensitive to Hdy-7-induced cytotoxicity, highlighting its translational potential. Another key regulator, progranulin (PGRN), a secreted growth factor involved in lysosome biogenesis and tumor progression, is reduced in several neurodegenerative diseases, including AD and PD. Restoring PGRN levels—through AL001 monoclonal antibody therapy, gene therapy, or recombinant PGRN replacement—has demonstrated therapeutic benefit [127].

Interestingly, not all therapeutic strategies rely on the upregulation of autophagy. Autophagy modulation in cancer therapy offers another promising avenue. Blocking autophagy or inducing lysosomal membrane permeabilization (LMP) can suppress tumor growth [128,129]. Autophagy serves as a fundamental adaptive process that sustains tumor cell survival under adverse microenvironmental stresses, including oxygen deprivation and nutrient scarcity. By recycling intracellular components, this catabolic mechanism provides metabolic flexibility and resilience. Inhibition of autophagy has therefore emerged as a compelling therapeutic approach to enhance the susceptibility of malignant cells to anticancer treatments. Pharmacological suppressors such as chloroquine (CQ), 3-methyladenine (3-MA), and bafilomycin A1 (BafA1) have demonstrated anticancer activity when applied independently or synergistically with established chemotherapeutic regimens. Activation of KRAS, a defining feature of pancreatic and several other cancers, has been closely linked to enhanced autophagy. Pharmacologic or genetic inhibition of the RAS–ERK signaling cascade often induces autophagy, which in turn supports tumor cell survival. Preclinical investigations indicate that concurrent inhibition of autophagy and downstream MAPK/ERK1/2 signaling substantially curtails tumor initiation and progression [130,131,132]. KRAS signaling also drives BRAF activation—a kinase commonly mutated in melanoma, gliomas, and colorectal carcinomas. Notably, in vemurafenib-resistant brain tumors, pharmacologic disruption of autophagy can reverse kinase inhibitor resistance, with clinical observations showing improved outcomes upon the addition of chloroquine to vemurafenib therapy [133,134]. In head and neck carcinoma (HNC) cell models, 3-MA was found to potentiate the cytotoxic effect of RITA by concomitantly repressing autophagy and antioxidant defenses, thereby overcoming drug resistance [135]. Likewise, sub-cytotoxic doses of BafA1 have been shown to selectively induce mitochondrial damage and apoptosis by preventing autolysosome formation in pediatric B-cell acute lymphoblastic leukemia cells [136]. Beyond these established inhibitors, ULK1 inhibitors (SBI-0206965, ULK101, MRT403), VPS34 inhibitors (VPS34-IN1, SAR405), and ATG4B-targeted compounds, which interfere with distinct stages of autophagy initiation and maturation, have also attracted much attention. But they have not yet entered clinical trials [137,138,139,140,141]. In addition, Baicalin inhibits non-small-cell lung cancer (NSCLC) proliferation by activating the lysosomal Ca^2+^ channel MCOLN3, disrupting Ca^2+^ homeostasis, and impairing autolysosomal degradation [142]. LMP represents a pivotal event linking lysosomal biology to cell death regulation. Upon LMP, lysosomal proteases—particularly cathepsins B, D, and L—are released into the cytosol, where they trigger downstream cell death pathways [143]. Siramesine, an σ-2 receptor ligand, has been shown to eliminate the pancreatic cancer stem cell population in mice implanted with tumors by inducing LMP [144]. Likewise, the synthetic flavonoid FV-429 disrupts lysosomal function and autophagic flux in T-cell malignancies, inducing cathepsin-mediated, caspase-independent cell death (CICD) and sensitizing cancer cells to chemotherapy [145].

Given that many disorders stem from substrate accumulation due to lysosomal dysfunction, diverse lysosome-targeted therapeutic approaches have been developed, ranging from modulation of the autophagy–lysosome system to direct restoration of lysosomal function and emerging protein degradation technologies. Restoring lysosomal acidification is a key therapeutic objective. For instance, aberrant LRRK2 kinase activity disrupts lysosomal function and increases PD risk; the small-molecule inhibitor DNL201 restores lysosomal activity [146]. In AD, loss of presenilin-1 (PSEN1) impairs V-ATPase V0a1 delivery, reducing lysosomal acidification. β_2_-adrenergic agonists such as isoproterenol reacidify lysosomes in familial AD fibroblasts by restoring ClC7 trafficking, thereby normalizing autophagic flux [147]. In addition, excessive ER–RyR–Ca^2+^ release contributes to lysosomal alkalinization in AD, which can be corrected with RyR inhibitors. Several small molecules—including C381, EN6, cyclic AMP analogs, NKH-477, SF-22, tetrandrine, nicotinamide riboside, curcumin analog C1, and PF11—also promote lysosomal acidification and functional recovery [12,148,149,150]. Beyond small molecules, nanomedicine-based strategies are emerging. Poly(lactic-co-glycolic acid) (PLGA) acidic nanoparticles restore lysosomal pH and proteolytic function in PD and AD models by reacidifying defective lysosomes. In Drosophila, feeding larvae with acidic nanoparticles partially corrects lysosomal pH defects, though dosage optimization is required to mitigate toxicity [151,152,153]. Ferroptosis is a distinct, regulated form of cell death driven by iron-dependent lipid peroxidation (LPO) and has emerged as a promising therapeutic target in cancer and neurodegenerative diseases. Recent findings highlight the lysosome as a central mediator of ferroptotic signaling; it coordinates both the initiation and execution phases of ferroptosis [154]. Lysosomal lipid peroxidation represents an early and decisive event that provokes iron efflux and amplifies cytosolic LPO through increased LMP and protease leakage. In ferroptosis-resistant cells, this lysosomal response is absent, whereas pharmacological disruption of lysosomal stability with chloroquine restores LMP and reinstates susceptibility to ferroptotic death [155]. Impaired lysosomal function disrupts lipid metabolic homeostasis and iron storage, resulting in either hyperactivation or suppression of ferroptosis. In neurons, for example, loss of the lysosomal protein prosaposin (PSAP) leads to aberrant lipid degradation, accumulation of reactive iron species, and ferroptotic neurodegeneration [156]. Therapeutic modulation of this pathway offers context-specific benefits. Ferroptosis inhibition can limit oxidative damage and enhance neuronal survival, while lysosome-targeted activation of ferroptosis may prove advantageous in cancer therapy. Small molecules that manipulate lysosomal iron have therefore attracted growing interest. Among them, Fentomycin-1 selectively mobilizes lysosomal iron and induces ferroptosis in iron-rich, CD44-high cancer cells [99], underscoring the potential of exploiting lysosomal iron metabolism as a new anticancer strategy.

Recent innovations in targeted protein degradation harness lysosomal pathways. Lysosome-targeting chimeras (LYTACs) exploit receptor-mediated endocytosis to degrade extracellular and membrane proteins, while autophagy-based technologies—including autophagy-targeting chimeras (AUTACs), autophagosome-tethering compounds (ATTECs), and chaperone-mediated autophagy (CMA)-based degraders—enable selective clearance of intracellular proteins and organelles. For example, LYTACs use dual-binding molecules to link target proteins with cell-surface lysosomal receptors such as CI-M6PR, directing them for degradation [157]. AUTACs employ guanine-derived tags to trigger S-guanylation and recruit damaged proteins or organelles to autophagosomes for degradation [158], whereas ATTECs directly tether target proteins to LC3 on autophagosome membranes, mediating ubiquitin-independent degradation [159]. CMA-based degraders incorporate a CMA-targeting motif that facilitates lysosomal delivery of specific cytosolic proteins, such as α-synuclein [160].

Finally, the dynamic interaction between mitochondria and lysosomes is crucial for sustaining cellular homeostasis. Urolithin A (UA), a gut microbiota-derived metabolite, restores mitochondrial and lysosomal homeostasis by normalizing cathepsin Z activity in AD, underscoring the potential of lysosomal modulation in neurodegenerative disease treatment [161].

**Table 1:** Small-molecule drugs: target the autophagy–lysosome system in neurodegenerative diseases and cancer.

Target	Drug Name	Disease	Most Advanced Clinical Trials	NCT No./References	
mTOR	Rapamycin(Sirolimus)	Lymphangioleiomyomatosis	FDA-approved	NCT00414648	[116,117]
		Lung cancer, Kidney cancer, Breast cancer, Head and neck squamous cell carcinoma	Preclinical	-	
	Temsirolimus	Advanced renal cell cancer	FDA-approved	NCT00065468	
	Everolimus	Renal cell cancer	FDA-approved	NCT00410124	
		Pancreatic neuroendocrine tumors		NCT00510068	
		Breast cancer		NCT00863655	
		Epilepsy		NCT01713946	
		Subependymal giant cell astrocytomas		NCT00789828	
	Ridaforolimus	Sarcomas	III	NCT00538239	
	GDC-0980	Renal cell cancer	II	NCT01442090	
		Endometrial cancer	II	NCT01455493	
		Breast cancer	I	NCT01254526	
AMPK	Metformin	Type 2 diabetes	FDA-approved	NCT02252965	[118,119]
		Colorectal cancer	III	NCT05921942	
		Breast cancer	III	NCT01101438	
		AD	II	NCT01965756	
TFEB	Celastrol	AD, Gastric, Renal cell carcinoma	Preclinical	-	[122,123]
	Trehalose	PD, HD			[124]
	Curcumin analog C1	AD			[148,149,150]
	PF11	AD			
PARP1	Poly (ADP-ribose) polymerase 1 (PARP1) inhibitors	AD, PD	Preclinical	-	[125]
		Ovarian cancer	FDA-approved	NCT01844986	
		Breast cancer		NCT01945775	
		Pancreatic cancer		NCT02184195	
		Prostate cancer		NCT05457257	
TrxR1/2	Hdy-7	Hepatocellular carcinoma, Colorectal cancer, Lung cancer	Preclinical	-	[126]
PGRN	AL001	AD	I/II	NCT05363293	[127]
Lysosomal lumen alkalizer	Chloroquine (CQ)	Glioblastoma multiforme	III	NCT00224978	[133,134]
		Malaria	FDA-approved	NCT01814423	
ATG4B	UAMC-2526	Colorectal cancer	Preclinical	-	[137,138,139,140,141]
	NSC185058	Osteosarcoma tumors		-	
	S130	Colorectal cancer		-	
ULK1	SBI-0206965	Renal cell cancer, Neuroblastoma	Preclinical	-	
	ULK101	Advanced cancers		-	
VPS34	VPS34-IN1	Breast cancer, Acute myeloid leukemia, Non-small-cell lung cancer	Preclinical	-	
	SAR405	Kidney cancer, Melanoma, Colorectal cancer, Central nervous system tumors		-	
	3-methyladenine (3-MA)	Colorectal cancer, Head and neck cancer		-	[135]
Lysosomal Ca^2+^ channel MCOLN3	Baicalin	Non-small-cell lung cancer	Preclinical	-	[142]
σ-2 receptor	Siramesine	Pancreatic cancer	Preclinical	-	[144]
		Small-cell lung cancer		-	
Lysosomal membrane	FV-429	T-cell malignancies	Preclinical	-	[145]
	Tetrandrine	AD		-	[148,149,150]
LRRK2	DNL201	PD	I	NCT03710707	[146]
ClC7	Isoproterenol (ISO)	AD	Preclinical	-	[147]
		Atrioventricular Block, Shock	FDA-approved	-	
V-ATPase	Bafilomycin A1 (BafA1)	Leukemia, Glioblastoma	Preclinical	-	[136]
	C381	AD			[148,149,150]
	NKH-477	AD			
TRPML1	SF-22	AD	Preclinical		[148,149,150]
SIRT1/SIRT3	Nicotinamide riboside (NR)	AD	II	NCT05617508	[148,149,150]
		PD		NCT06853743	
		Breast cancer	II	NCT05732051	
Cathepsin Z(Ctsz)	Urolithin A (UA)	AD	Preclinical	-	[161]
		Prostate cancer	II	NCT06022822	

## 7. Conclusions and Perspectives

The accumulation of misfolded and aggregation-prone proteins such as amyloid-β/tau, α-synuclein, and mutant huntingtin is a defining pathological hallmark of many neurodegenerative disorders. These toxic aggregates disrupt proteostasis, compromise organelle integrity, and perturb multiple homeostatic networks. Although neurodegenerative diseases arise from distinct molecular etiologies, their pathological consequences converge on lysosomal dysfunction. Aggregated proteins can physically impede autophagic flux, elicit oxidative and nitrosative stress, disrupt inter-organelle communication (e.g., mitochondria–lysosome contacts), and interfere with transcriptional regulators such as TFEB. Collectively, these insults impair lysosomal acidification and degradative capacity, resulting in defective lysosomal pH regulation, impaired autophagosome–lysosome fusion, enzymatic deficiencies, and lysosomal membrane permeabilization triggered by oxidative damage, lipid peroxidation, or aggregate-induced stress. These defects hinder the clearance of toxic cargo, establishing a self-amplifying cycle of proteotoxicity that drives progressive neuronal loss. A group of genes—such as APOE4, PSEN1, LRRK2, PINK1, and Parkin—has been associated with neurodegenerative diseases. These genes play critical roles in the autophagy–lysosomal pathway, acting at different stages of the autophagic process. Thus, neurodegenerative diseases can be mechanistically categorized according to the principal site of dysfunction within the autophagy–lysosome pathway. Disorders such as Huntington’s disease exhibit impaired autophagy initiation; Parkinson’s disease usually displays defects in autophagosome maturation, whereas BPAN and Alzheimer’s disease are characterized primarily by defective autophagosome–lysosome fusion. Advanced Parkinson’s disease often culminates in profound lysosomal degradation failure, reflecting end-stage collapse of proteolytic capacity.

Pharmacological modulation of the autophagy–lysosome axis holds substantial promise in both neurodegenerative diseases and cancer, yet the lysosomal system assumes fundamentally opposing roles in these settings. In neurodegeneration, autophagic activity is typically suppressed, leading to the accumulation of aberrant proteins and damaged organelles that precipitate neuronal toxicity and death. Accordingly, therapeutic strategies focus on enhancing lysosomal function or restoring autophagic flux—for example, through mTORC1 inhibition, TFEB activation, or restoration of lysosomal acidification. By contrast, tumor cells often exploit the autophagy–lysosome machinery to endure metabolic stress, hypoxia, and therapeutic challenges. Hence, anticancer interventions aim to disrupt lysosomal integrity, for instance by inducing LMP or blocking autophagic flux using agents such as baicalein or FV-429, thereby provoking metabolic collapse and apoptosis. Beyond opposing autophagic flux, the structural and functional nature of lysosomal alterations also diverges between these diseases. In neurodegeneration, lysosomes exhibit defective acidification, diminished hydrolase activity, and membrane fragility—culminating in degradative failure. In cancer, lysosomal remodeling involves altered subcellular localization, enhanced exocytosis, and a reprogrammed membrane lipid composition, which facilitates invasion and metastasis rather than impairing degradation. Additionally, these contrasts underscore the necessity for disease-specific strategies when therapeutically targeting the autophagy–lysosome network.

Continued elucidation of autophagy–lysosome regulation is driving the development of pharmacological modulators targeting discrete nodes of this pathway. In cancer, autophagy inhibitors such as chloroquine and hydroxychloroquine have entered clinical evaluation—often in combination with chemotherapeutic, targeted, or immunotherapeutic regimens—while the mTOR inhibitor temsirolimus has gained FDA approval for advanced renal cell carcinoma. Nevertheless, key challenges remain. Many autophagy-modulating compounds lack sufficient specificity, risking off-target effects. Drug delivery to the brain is hindered by the blood–brain barrier, and while nanocarriers such as liposomes and nanoparticles have improved CNS delivery, current interventions primarily alleviate symptoms rather than provide cures. Equally critical is the absence of robust real-time biomarkers to monitor lysosomal pH, hydrolase activity, and autophagic flux—parameters essential for evaluating therapeutic responses. Future research should focus on identifying selective regulators of the autophagy–endolysosomal system and on refining drug-delivery strategies. Moreover, in cancer, the autophagy–lysosome pathway exhibits a context-dependent, dual role; thus, precise interventions tailored to tumor type, stage, and microenvironment are needed to exploit the benefits of autophagy modulation without inadvertently promoting tumorigenesis. Aging is accompanied by a rising burden of both cancer and neurodegenerative diseases. Given the molecular crosstalk between cancer and neurodegenerative disorders, advances in one field are likely to benefit the other. Elucidating the cellular and molecular mechanisms that underlie their shared and divergent pathways will be crucial for identifying new therapeutic targets. Such cross-disease strategies have the potential not only to improve outcomes in cancer and neurodegeneration separately but also to address the growing challenge of multimorbidity in aging populations.

## Data Availability

No new data were created or analyzed in this study. Data sharing is not applicable to this article.
